# Hypomethylation of miR-142 promoter and upregulation of microRNAs that target the oxytocin receptor gene in the autism prefrontal cortex

**DOI:** 10.1186/s13229-015-0040-1

**Published:** 2015-08-14

**Authors:** Michal Mor, Stefano Nardone, Dev Sharan Sams, Evan Elliott

**Affiliations:** Bar Ilan University Faculty of Medicine, Hanrieta Sold 8, Safed, 13215 Israel

**Keywords:** Autism, MicroRNA, DNA methylation, Epigenetics, Oxytocin receptor

## Abstract

**Background:**

MicroRNAs are small RNA molecules that regulate the translation of protein from gene transcripts and are a powerful mechanism to regulate gene networks. Next-generation sequencing technologies have produced important insights into gene transcription changes that occur in the brain of individuals diagnosed with autism spectrum disorder (asd). However, these technologies have not yet been employed to uncover changes in microRNAs in the brain of individuals diagnosed with asd.

**Methods:**

Small RNA next-generation sequencing was performed on RNA extracted from 12 human autism brain samples and 12 controls. Real-time PCR was used to validate a sample of the differentially expressed microRNAs, and bioinformatic analysis determined common pathways of gene targets. MicroRNA expression data was correlated to genome-wide DNA methylation data to determine if there is epigenetic regulation of dysregulated microRNAs in the autism brain. Luciferase assays, real-time PCR, and Western blot analysis were used to determine how dysregulated microRNAs may regulate the expression and translation of an autism-related gene transcript.

**Results:**

We determined that miR-142-5p, miR-142-3p, miR-451a, miR-144-3p, and miR-21-5p are overexpressed in the asd brain. Furthermore, the promoter region of the miR-142 gene is hypomethylated in the same brain samples, suggesting that epigenetics plays a role in dysregulation of microRNAs in the brain. Bioinformatic analysis revealed that these microRNAs target genes that are involved in synaptic function. Further bioinformatic analysis, coupled with in vitro luciferase assays, determined that miR-451a and miR-21-5p can target the oxytocin receptor (OXTR) gene. OXTR gene expression is increased in these same brain samples, and there is a positive correlation between miR-21-5p and OXTR expression. However, miR-21-5p expression negatively correlates to production of OXTR protein from the OXTR transcript. Therefore, we suggest that miR-21-5p may attenuate OXTR expression in the human autism brain.

**Conclusions:**

Our data suggests that dysregulation of microRNAs may play a biological role in the brain of individuals of autism. In addition, we suggest an interaction between epigenetic mechanisms and microRNA dysregulation in the brain. Overall, this data adds an important link in our understanding of the molecular events that are dysregulated in the brain of individuals diagnosed with autism.

**Electronic supplementary material:**

The online version of this article (doi:10.1186/s13229-015-0040-1) contains supplementary material, which is available to authorized users.

## Background

Autism spectrum disorder (asd) is a neurodevelopmental disorder that includes disruptions in social behavior, communication, and stereotypic behaviors [1]. While a plethora of scientific research has indicated that both genetic and unknown factors have primary roles in the etiology of autism [2, 3], there is still scarce knowledge of the precise molecular mechanisms that are responsible for autistic behavior.

Recent studies have focused on molecular changes in the frontal cortex of individuals diagnosed with autism. Voineagu et al., using a whole genome transcriptome approach, determined the downregulation of many transcripts encoding synaptic proteins and the overexpression of many transcripts encoding immune system proteins in the frontal cortex of individuals with autism [4]. A separate whole genome RNA sequencing study recently found a similar overexpression of immune system-related gene transcripts in a separate autism brain cohort [5]. Using a whole genome approach, we have previously demonstrated epigenetic differences on genomic regions responsible for synaptic transmission and immune regulation in the frontal cortex of individuals with autism [6]. Therefore, gene transcription dysregulation in the brain of individuals diagnosed with asd may partially be due to epigenetic modifications. However, there are several other gene transcription and translation regulatory pathways that have yet to be probed for dysregulation in the brain of individuals with asd. Research into these mechanisms is necessary in order to produce a complete picture of molecular dysregulation in the brain of individuals with asd.

MicroRNAs are small regulatory nucleotides that inhibit translation of target messenger RNAs. MicroRNA are approximately 21 bases, and form complexes with the RNA-induced silencing complex (RISC), which includes Dicer and a member of the Argonaute family of proteins [7]. The RISC-microRNA complex then binds target messenger RNAs that are complementary at the “seed region” of the microRNA, typically nucleotides 2–8 [8]. The binding to messenger RNAs will lead to decreased protein translation, either through degradation of the target mRNA or the inhibition of protein translation through compartmental storage of mRNA complexes [9, 10]. While it is yet to be determined the exact number of microRNAs that are expressed in the human genome, there are currently up to 1881 putative human microRNA precursors and 2588 putative mature microRNAs in the miRBase database [11], and each microRNA may target multiple mRNAs. Therefore, microRNA upregulation or downregulation may influence entire protein networks, and is a uniquely powerful regulatory mechanism.

Previous research has determined dysregulation of microRNAs in the brain in various psychiatric conditions [12], including schizophrenia [13] and Huntington’s disease [14]. Dysregulation of serum microRNAs have been detected in individuals with autism [15]. The authors uncovered a list of dysregulated microRNAs that target mRNAs involved in the biological process of axon guidance. One previous study looked at differences in microRNAs in the human autism brain using microarray technology [16]. They did not detect any microRNAs that were differentially expressed between control and autism brains, although they detected several microRNAs that had a higher variation of expression in the autism cohort. However, no study has previously been reported on dysregulation of microRNAs in the human autism brain by using next-generation sequencing technologies, which are capable of detecting all microRNAs in the sample, and have a significantly higher sensitivity. In this current work, using next-generation sequencing followed by real-time PCR, we determined significant upregulation of multiple microRNAs in the brain of individuals with asd. Further analysis revealed that microRNA upregulation may have a very relevant impact on biological pathways involved in autism, both by targeting oxytocin receptor expression and targeting several gene ontology pathways that are relevant to autism, including synapse function and signal transduction. In addition, we suggest a connection between DNA methylation dysregulation and the overexpression of miR-142 in autism. Overall, our data provides evidence for a role of brain microRNA dysregulation in the biology of autism spectrum disorders.

## Methods

### Brain tissue samples

Brain tissue samples from 12 autism cases and 12 controls were obtained from the Autism Tissue Program (ATP). All of these samples were received from the Harvard Brain Bank except for two brain samples of individuals with asd, which were obtained from the UK Brain Bank for Autism (University of Oxford). Brodmann’s area 10 was used in this analysis, and information about samples is found in Additional file 1. Full clinical information about the individuals, when available, is provided upon request (http:www.autismtissueprogram.org). The experimentation on these samples was given ethical approval through the Bar Ilan University Institutional Review Board (Helsinki Committee), and patient or parental consent was given to the Autism Tissue Program for all individuals tested in this study.

### Total RNA extraction and generation of small RNA libraries

Total RNA was extracted from approximately 70 mg of liquid nitrogen pulverized tissue using the miRNeasy kit and treated with RNase-Free DNase (Qiagen). For each brain sample, 1 μg of RNA was used to construct sequencing libraries using Illumina’s TruSeq Small RNA Sample Prep Kit, according to the manufacturer’s protocol (Illumina, San Diego, CA). In brief, small RNA molecules were adapter-ligated, reverse transcribed, PCR amplified, and gel purified to generate the library. Multiplexed samples were equimolarly pooled into sets of 24 samples per flow cell lane and sequenced using 1 × 50 bp single-end reads on Illumina’s MiSeq system at Bar Ilan University Faculty of Medicine sequencing core facility. Samples were sequenced twice to obtain a high read count. Demultiplexing and FASTQ file generation (raw sequence read plus quality information in Phred format) were done using Illumina’s Consensus Assessment of Sequence and Variation (CASAVA) pipeline.

### Alignment of miRNA-seq reads and differential expression analysis

FASTQ files were uploaded to the miRanalyzer web-based tool for mapping small RNAs to the miRBase database. miRanalyzer uses the short-read aligner bowtie to align to the reference database [17]. Parameters used included a minimum match seed length of 17 base pairs with a maximum of one mismatch within this region. After identifying all possible matches with a minimum 17 base pair match, the longest match was chosen as the identified microRNA. This produced small RNA transcription maps, with read counts, for each sequencing library. Normalized read counts were created using DESeq [18], where a scaling factor was computed as the median of the ratio, of each microRNA, of its read count over its geometric mean among all samples. Each sample’s count was then divided by this scaling factor. The normalized reads can be found in Additional file 2. To analyze for differential expression between the different experimental groups, the transcription maps were loaded onto the miRanalyzer differential expression tool. This tool is based on the DESeq package, which produces a differential expression for each identified microRNA, with the appropriate fold changes, *p* value, and FDR-adjusted *p* value.

### microRNA quantitative PCR

Total RNA from each sample was reverse transcribed with the miScript II RT Kit. Real-time PCR was then performed with the miScript SYBER Green PCR kit, according to the manufacturer’s protocols. A specific forward primer was used for each microRNA, together with a universal reverse primer. Specific primers used are in Additional file 3. U6 snRNA was used as the housekeeping gene in these experiments. All PCR reactions were performed on the ViiA™ 7 Real-Time PCR System. Real-time PCR reactions were performed on all samples used in the sequencing, excluding one autism individual (UK20119, Additional file 1: Table S1), which we only had enough RNA sample for sequencing.

### Methylation pyrosequencing

Pyrosequencing technology was used to further validate differences in DNA methylation detected with Infinium HumanMethylation450 BeadChip. We tested three CpG sites in the promoter of microRNA-142. DNA samples were submitted to the School of Medicine and Dentistry Genome Centre Barts and London (London, UK) and processed by the following procedure. Briefly, 500 ng DNA from each individual was treated with sodium bisulphite using the EZ96-DNA methylation kit according to the manufacturer’s recommendation, and amplified by a bisulphite-polymerase chain reaction. Quantitative DNA methylation analysis of each CpG was conducted using PSQ96 Pyrosequencer (Qiagen, Valencia, CA, USA) [19]. In short, a biotinylated PCR product was created from the genomic region and hybridized to a sequencing primer. The PCR product was incubated with DNA polymerase, luciferase, ATP sulfurylase, APS, and luciferin. After the addition of a nucleotide to the reaction, the release of a diphosphate group will induce the generation of light, which is read by the Pyrosequencer. The amount of light relates to the amount of that particular nucleotide found at that genomic region. For a more detailed protocol, please refer to Kreutz et al. [19]. All samples were analyzed, excluding AN12137 (control sample, Additional file 1: Table S1), because DNA from this sample did not pass the pyrosequencing quality control checks.

### Messenger RNA real-time PCR

Real-time PCR was performed on an ABI ViiA™ 7 Real-Time PCR detection system in 10 μl volume containing FastStart Universal SYBR Green Master (Roche) and primers at a concentration of 0.250 μM each. All primers used were designed using Primer-BLAST and tested for the efficiency through a standard curve. All primer sequences are listed in Additional file 3. The strategy used for the normalization of quantitative RT-PCR data from human genes was geometric averaging of multiple internal control genes according to the Vandesompele et al.’s method [20]. We used three housekeeping genes (*GAPDH*, *HPRT1*, and *POLR2a*) that represent an accurate control for mRNA expression analysis of postmortem brain samples. For each housekeeping gene, we measured the gene stability (M value) and ranked it using the geNorm algorithm.

### Construction of psiCHECK2-3′UTR construct for luciferase assays

Oxytocin receptor (OXTR) 3′ UTR sequence was PCR amplified from human genomic DNA (primers: 5′-TTCCACAGCATCAAGCAGTC-3′ and 5′-CCCAGCAGAGTGAACGTCTT-3′). PCR fragment was digested with Not1 and Xho1 and ligated into the psiCHECK2 reporter plasmid (Promega), which had been cut with the same restriction enzymes. The 3′ UTR of OXTR was cloned directly downstream of the Renilla luciferase gene in the psiCHECK2 plasmid, which also contains the Firefly luciferase gene as an internal control. Cloning orientation was verified by sequencing. Plasmids expressing miR-451a, miR-21-5p, and miR-7 and plasmid expressing only GFP were purchased from OriGene (Rockville, MD, USA). Plasmids expressing microRNAs also express GFP.

### Transfections and luciferase assay in HEK293T cells

Cells were grown in a 24-well format to a 70–85 % confluency and transfected using polyethyleneimine (Sigma, St. Louis, MI, USA) with the following plasmids: 100 ng of psiCHECK2-3′ UTR plasmid and 400 ng of pEGFP– miR-451a, pEGFP– miR-21-5p, pEGFP– miR-7, or empty pEGFP overexpression plasmids. At 72 h after transfection, cells were lysed, and luciferase reporter activity was assayed as described previously [21]. Renilla luciferase values were normalized to control firefly luciferase levels and averaged across three-well repetitions per condition. Data presented are the average of three experiments.

### Western blot

Brain tissue was homogenized in a tissue lysis buffer containing 50 mM Tris-HCl (pH 7.5), 150 mM KCl, and 0.32 M sucrose supplemented with protease inhibitor cocktail (Sigma). Protein estimation was done with Bradford reagent (Sigma, St. Louis, MI, USA). Samples (20 μg) were subjected to SDS-PAGE and transferred onto a nitrocellulose membrane. The membrane was blocked for 1 h in PBS with Tween 20 and 5 % non-fat milk followed by overnight incubation with a primary antibody in 5 % BSA. The primary antibodies used were the following: anti-OXTR (1:1000 R&D Systems, Minneapolis, MS, USA) and anti-Hsc70 rabbit serum (1:1000, previously described [22]). Following washing, the membranes were incubated with LI-COR dye-conjugated secondary antibody for 1 h. Membranes were then scanned on the LI-COR Odyssey scanner. The intensity of the bands was quantified using LI-COR imaging software, and OXTR protein levels were normalized against the Hsc70 protein levels.

### Statistics

All statistical analyses, including *t* tests and Spearman’s correlation analysis, on real-time PCR and pyrosequencing methylation data were performed with SPSS software package (version 20.0; SPSS, Chicago, IL, USA). We used two-tailed independent *t* test for groups with equal variance of distribution, unless otherwise noted.

## Results

### Identification of differentially expressed microRNAs in the brain of individuals diagnosed with asd

In order to determine changes in microRNA expression in the brain of individuals diagnosed with autism, we studied postmortem brain samples from 12 individuals with an ADIR-R-confirmed diagnosis of autism and 12 matched controls. All samples were from Brodmann area 10, a subcomponent of the frontal cortex, which was previously examined for differences in DNA methylation between controls and individuals with autism [6]. Extensive information about these samples, including age and sex, are found in Additional file 1. There are no significant differences in age between the control and autism samples, and there is one female in the control group compared to two in the autism group. There is a relatively wide range of ages in both the control and autism groups, reflecting the current availability of human autism brain tissues. However, all brain samples are from adult individuals, therefore excluding the variability of working with tissues from different developmental time points. Following total RNA extraction and small RNA sequencing library preparation, the libraries were sequenced on the MiSeq Illumina high throughput sequencer. The miRanalyzer tool was used to map sequenced read and to identify microRNAs that were differentially expressed between the two experimental groups [17]. According to this initial analysis, 23 microRNAs were found to be differentially expressed between the control and autism groups (FDR < 0.05), including 18 microRNAs that were upregulated in the autism group, and five microRNAs that were downregulated in the autism group (Table 1).

**Table 1:** List of differentially expressed microRNAs according to whole RNA sequencing analysis

ID	Control mean	Autism mean	Fold change	pval	padj
hsa-miR-338-5p	195.14	866.47	4.44	5.47E−81	6.04E−78
hsa-miR-3168	1.79	16.51	9.23	3.07E−30	1.70E−27
hsa-miR-451a	797.35	1841.5	2.31	7.30E−20	2.69E−17
hsa-miR-21-5p	571.1	1304.5	2.28	1.34E−15	3.69E−13
hsa-miR-7-5p	131.07	309.53	2.36	4.85E−15	1.07E−12
hsa-miR-21-3p	20.9	60.5	2.89	3.40E−11	6.26E−09
hsa-miR-142-5p	9.69	26.67	2.75	2.03E−08	3.21E−06
hsa-miR-142-3p	3.68	10.97	2.98	2.34E−06	0.0003
hsa-miR-19a-3p	4.83	13.71	2.84	3.74E−06	0.0005
hsa-miR-211-5p	14.67	3.04	0.2071	4.12E−06	0.0005
hsa-miR-19b-3p	45.41	97.86	2.16	4.61E−06	0.0005
hsa-miR-219-5p	75.57	144.72	1.91	1.04E−05	0.001
hsa-miR-144-3p	2.25	7.73	3.43	1.25E−05	0.0011
hsa-miR-137	68.05	125.42	1.84	1.96E−05	0.0015
hsa-miR-34a-5p	81.74	47.38	0.5797	5.52E−05	0.0041
hsa-miR-146a-5p	72.06	121.44	1.69	8.27E−05	0.0054
hsa-let-376c-3p	23.28	44.75	1.92	8.00E−05	0.0054
hsa-let-7a-5p	18985.7	12852	0.6769	0.0004	0.0205
hsa-miR-379-5p	37	65.13	1.76	0.0003	0.0205
hsa-miR-92b-3p	2911.42	1957	0.6722	0.0008	0.0441
hsa-miR-3960	66.41	30.22	0.4551	0.0008	0.0441
hsa-miR-494	3.01	6.37	2.12	0.0009	0.0443
hsa-miR-155-5p	16.16	30.14	1.88	0.0009	0.0443

**Fig. 1:**
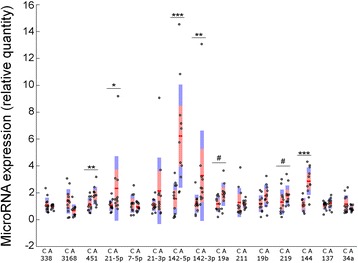
miR-451a, miR-142-5p, miR-142-3p, miR-144-3p, and miR-21-5p are overexpressed in the autism brain samples. Real-time PCR was performed on the first 15 microRNAs from the list in Table 1. The data is represented as box plots, with each *dot* representing an individual brain sample. The *line* identifies mean, and *red* and *purple* areas represent standard error of the means and standard deviation, respectively. In the *x*-axis, C = control and A = autism. Two-tailed students *t* tests *p < 0.05, **p < 0.01, ***p < 0.001. #*p* < 0.05 in one-tailed students *t* test

In order to identify individual microRNAs with a high confidence of dysregulation in the autism samples, we employed real-time PCR to detect the levels of the top 15 differentially expressed microRNAs in the same brain samples. Of these microRNAs we studied, real-time PCR analysis detected significant upregulation of five microRNAs (Fig. 1), including miR-142-5p (*p* = 0.0003), miR-142-3p (*p* = 0.003), miR-21 (*p* = 0.022), miR-451a (*p* = 0.007), and miR-144-3p (*p* = 0.0001). Two additional microRNAs were significantly upregulated when applying a one-tailed *t* test. The inability to validate more of the microRNAs found in the sequencing analysis may partly be a consequence of the relatively lower sensitivity and specificity of the real-time PCR method, in comparison to the sequencing method, although we cannot dismiss the possibility of false positives in the sequencing results. We note that dysregulation of a 5p form of the microRNA does not mean there is a dysregulation of the 3p form, and vice versa, which is due partly to the fact that both forms are not always expressed in the same tissue. All of our additional analysis was performed on the five microRNAs that are verified as upregulated using the two-tailed analysis.

### MicroRNA-142 overexpression correlates with decreased methylation of its promoter region in the brain of individuals diagnosed with asd

**Fig. 2:**
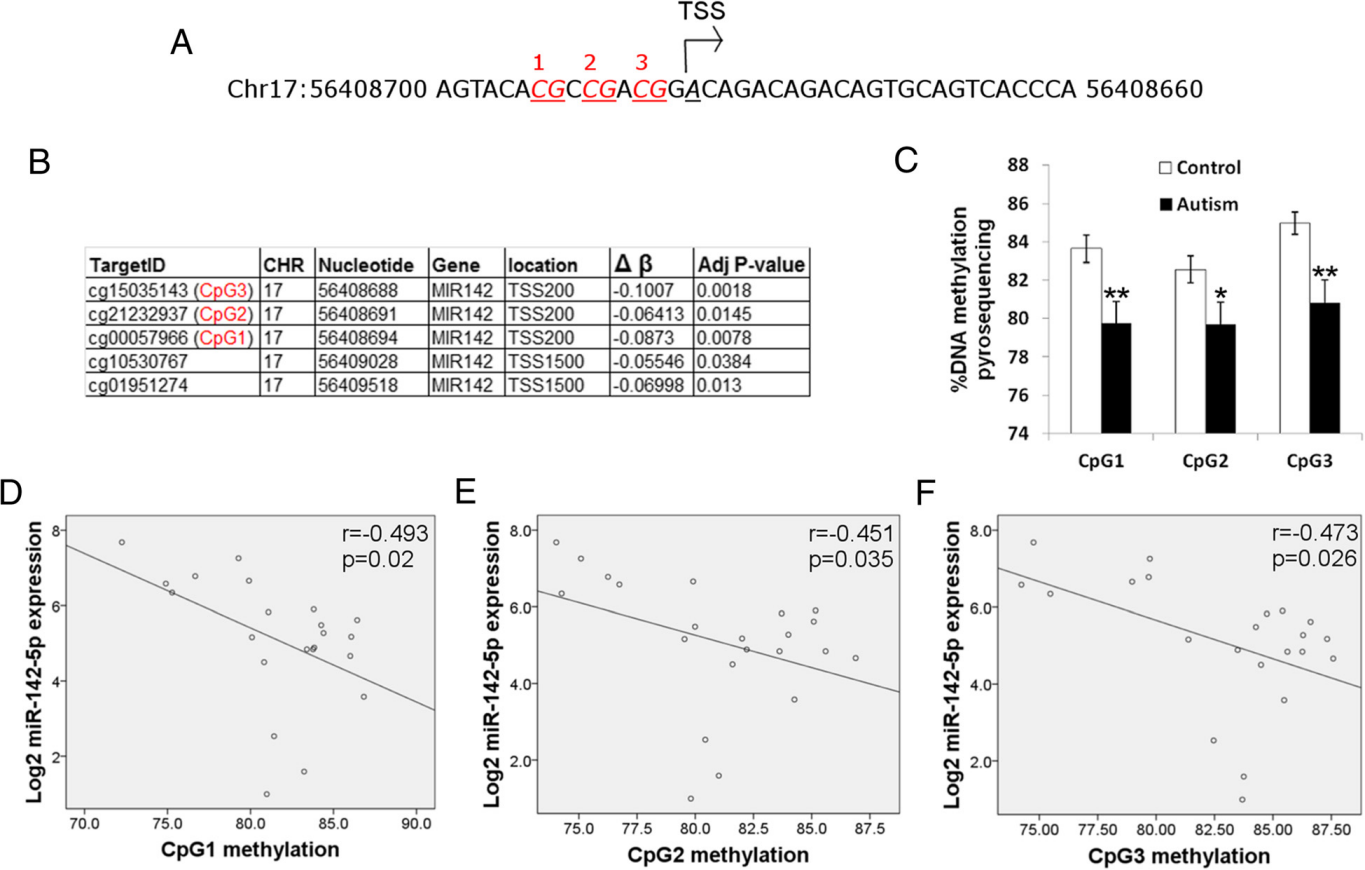
Promoter of miR-142 is hypomethylated in autism brain. **a** Scheme of nucleotide sequence directly surrounding the transcription start site (TSS) of miR-142. Three CG dinucleotides found directly upstream of TSS are highlighted in *red*. **b** DNA methylation microarray analysis of miR-142 gene, showing five CpGs that are significantly hypomethylated. **c** Pyrosequencing analysis of the three CpGs directly upstream of the TSS of miR-142. *p value <0.05, **p value <0.01. One-tailed *t* test. **d**–**f** Spearman’s correlation coefficient, including *p* value between miR-142-5p gene expression and DNA methylation. Each graph represents a correlation between miR-142-5p and a separate CpG site that was analyzed by pyrosequencing

In a previous study, we used the Illumina 450K methylation array to identify changes in DNA methylation at the genome-wide level in the same brain samples [6]. We compared our data from this current study to the list of differentially methylated CpG sites in the previous study. Of great interest, five CpGs in the promoter region of the miR-142 gene were hypomethylated in the autism brain samples, according to the microarray analysis (Fig. 2a, b). These include three CpGs that are found within ten nucleotides of the transcription start site for miR-142 (marked in red in Fig. 2a). No CpGs were dysregulated in the genetic regions encoding miR-21, miR-144, or miR-451 [6]. Subsequently, we used pyrosequencing, an independent method for quantifying DNA methylation, to determine the DNA methylation level of these three CpGs at the transcription start site (Fig. 2c). Pyrosequencing confirmed that there is a significant decrease of DNA methylation of these three CpGs in the autism brain. In addition, Spearman’s correlation analysis found a significant inverse correlation between the methylation levels at each specific CpG and expression levels of miR-142-5p (Fig. 2d–f) and miR-142-3p (Additional file 4). Therefore, we can directly correlate between decreased methylation of the miR-142 promoter and increased expression of the miR-142 transcripts in the brain samples of individuals diagnosed with asd. These results suggest a role for epigenetic dysregulation in miR-142 overexpression in the autism brain samples.

### Gene ontology analysis

**Fig. 3:**
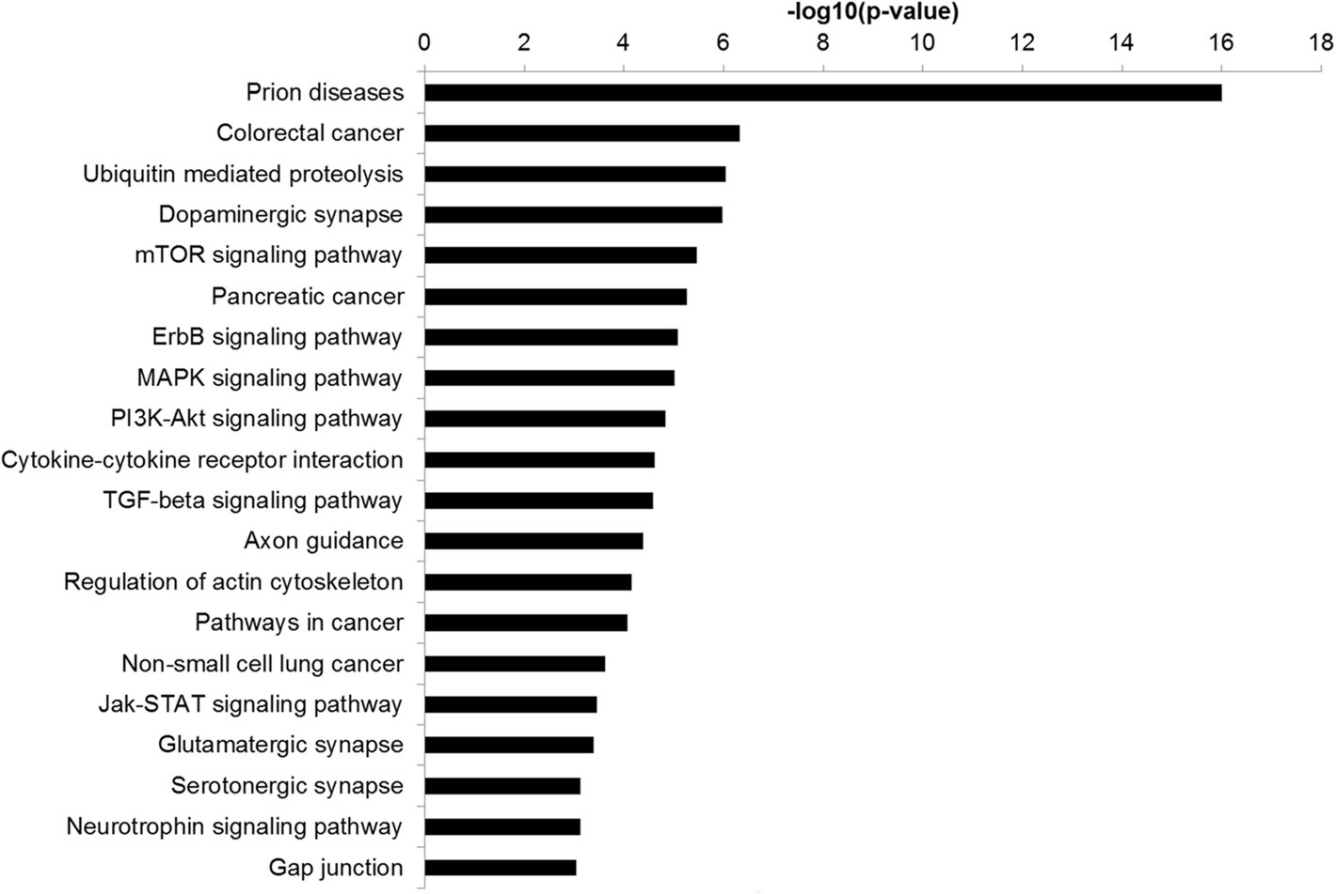
Gene ontology analysis of predicted targets of miR-142-5p, miR-142-3p, miR-21-5p, miR-144-3p, and miR-451a. Gene ontology analysis was performed by DIANA-lab software. Chart contains all gene ontology categories that are considered significant (adjusted *p* value <0.05)

To gain further insight into the biological meaning of the dysregulation of miR-142-5p, miR-142-3p, miR-21-5p, miR-144-3p, and miR-451a in the autism brain samples, we performed gene ontology analysis on the targets of these microRNAs, using the DIANA-lab software [23]. Several categories were enriched, particularly in categories related to signal transduction (mTOR signaling, ErbB signaling, MAPK signaling, etc.), synaptic categories (dopaminergic synapse, axon guidance, glutamatergic synapse, serotonergic synapse, neurotrophin signaling pathway), and many cancer-related categories (Fig. 3). Considering that recent data have implicated inflammatory processes in the etiology of autism [24–26], the gene ontology category of TGF-beta signaling may also be very relevant to asd.

### miR-21-5p and miR-451a target the oxytocin receptor gene

**Fig. 4:**
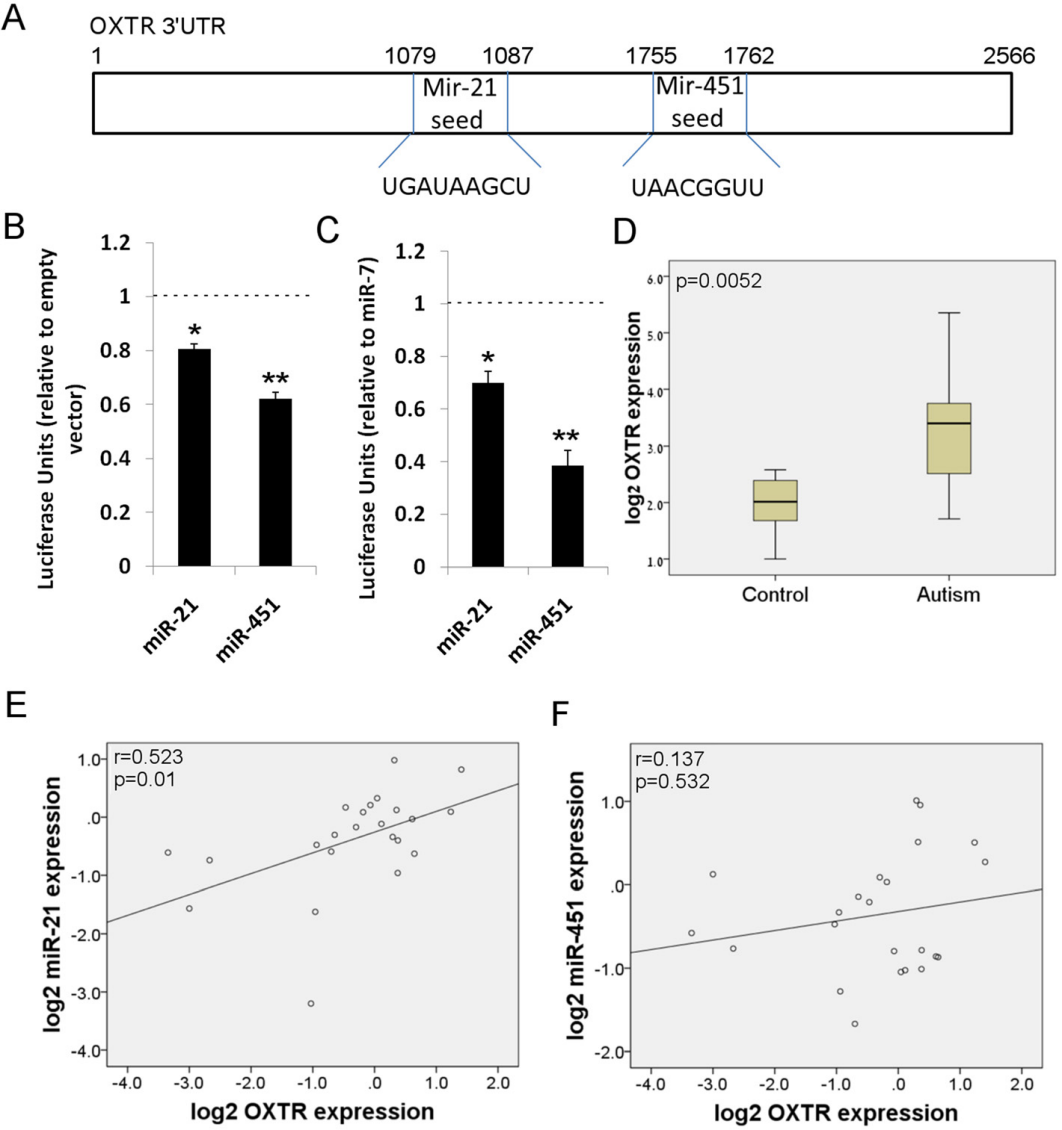
miR-451a and miR-21-3p target OXTR 3′UTR, and miR-21-5p expression levels correlate with OXTR expression levels. **a** Schematic representation of the 3′UTR of the OXTR mRNA, containing seed regions for miR-451a and miR-21-5p. **b** Luciferase assays assessing the effect of microRNA expression on the 3′UTR of the OXTR transcript. Expression of either miR-451a or miR-21-5p led to a significant reduction in translation from the luciferase-3′UTR OXTR transcript, compared to GFP expressing vector. *p value <0.05, **p value <0.01. Two-tailed *t* test. **c** Luciferase assay showing effect of miR-451a and miR-21-5p on luciferase expression, in comparison to miR-7. *p value <0.05, **p value <0.01. One-tailed *t*-test. **d** Box and whisker plot of OXTR gene expression levels in human brain, as assessed by real-time PCR. *p* = 0.0052 in two-tailed *t*-test. **e**, **f** Spearman’s correlation coefficient and respective *p* value between miR-21-5p (**d)** or miR-451a (**e**) expression and OXTR gene expression. There is a significant positive correlation between miR-21-5p expression and OXTR gene expression

To gain more insight into interesting individual mRNAs that are targets of more than one of our microRNAs, we employed the microRNA target resource at microrna.org [27]. Of great interest, the 3′UTR of the OXTR gene contains binding sites for seed sequences of microRNA-21 and microRNA-451. OXTR is one of the main molecular pathways that regulate mammalian social behavior [28], and genetic perturbations in OXTR have been implicated in subpopulations of individuals with asd [29], including Asperger’s syndrome [30]. There is an eight-nucleotide seed sequence for microRNA-21, starting at nucleotide 1079 of the 3′UTR of the OXTR transcript, while there is an eight-nucleotide seed sequence for miR-451a at nucleotide 1755 of the same 3′UTR (Fig. 4a). In order to experimentally determine if these microRNAs may directly target the OXTR 3′UTR, we performed luciferase assays. We constructed a plasmid that expresses the luciferase gene fused to the 3′UTR of the OXTR mRNA. This plasmid was cotransfected with a plasmid expressing GFP alone, GFP and miR-21-5p, or GFP and miR-451a. Both miR-21-5p and miR-451a induced a decrease in luciferase luminescence, compared to transfections with GFP alone (Fig. 4b). In a separate luciferase assay, we examined if miR-21-5p and miR-451a downregulate luciferase expression, in comparison to a microRNA that does not target OXTR, miR-7. In fact, miR-451a and miR-21-5p both significantly decreased luciferase expression, in comparison to miR-7 (Fig. 4c). This provides in vitro evidence that these two microRNAs can target the OXTR gene.

To gain more insight into the relationship between these microRNAs and OXTR expression, we performed real-time PCR to detect the levels of OXTR mRNA in the human brain samples. OXTR is significantly overexpressed in the autism brain samples (Fig. 4c; *p* = 0.005). In a previous analysis of DNA methylation levels in these same brain samples, there were no differences in the methylation of the OXTR gene, suggesting that the differences in OXTR gene transcription are not related to methylation [6]. While the increase of OXTR in the autism brain samples may not be intuitive, this finding validates a previous study [31] of increased OXTR expression in the frontal cortex of autistic individuals. Pearson’s correlation analysis reveals that there is a direct positive correlation between OXTR expression and the expression of microRNA-21 (Fig. 4d), but not miR-451a (Fig. 4e), in the brain samples from the current study. Therefore, miR-21-5p and OXTR expression is increasing in the same individuals.

**Fig. 5:**
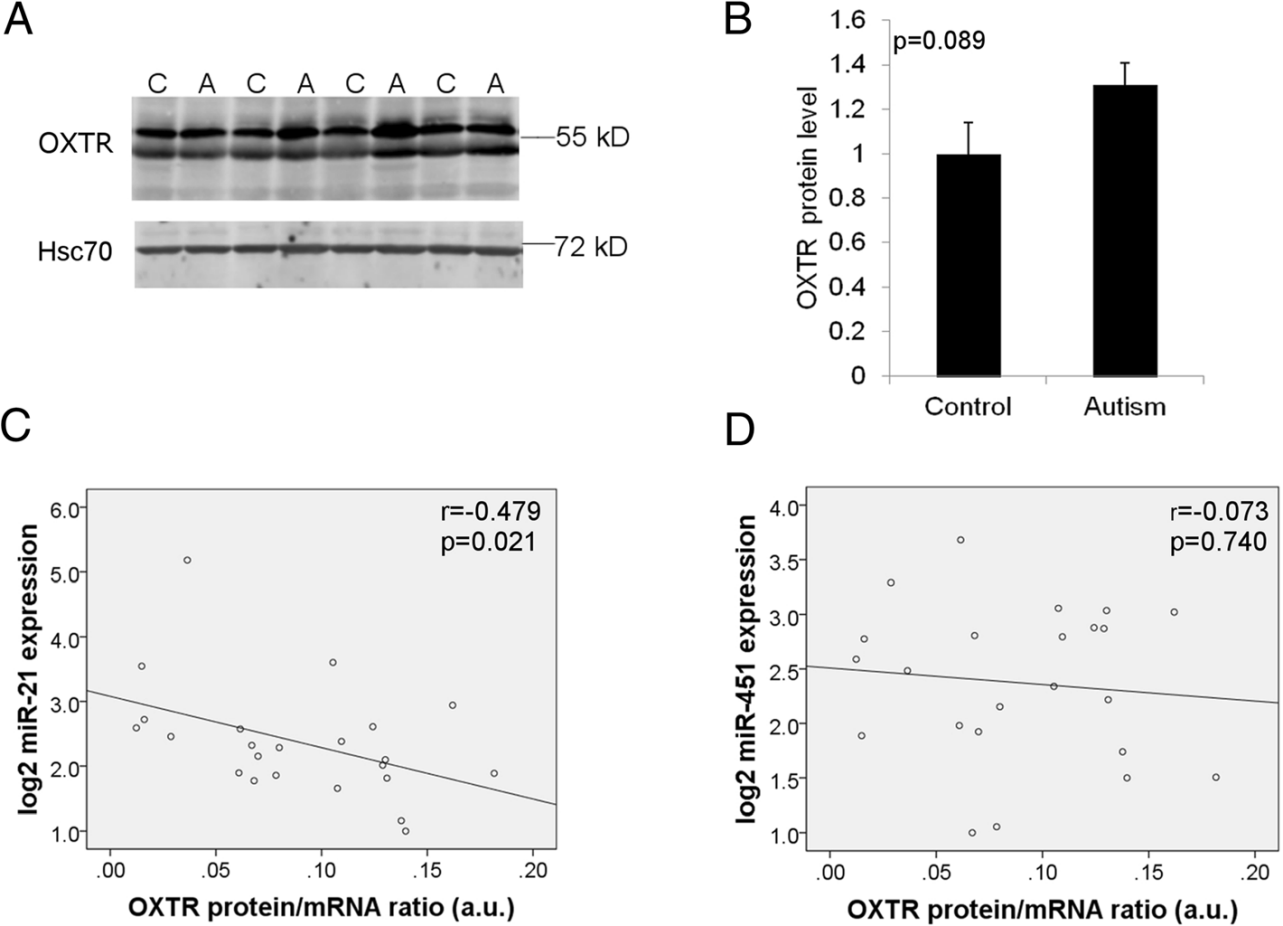
miR-21-5p expression correlates with less OXTR protein translation from mRNA in the brain. **a** Western blot analysis of OXTR in the human brain samples. Hsc70 is the endogenous control. **b** Quantitative analysis of relative OXTR protein levels in control and autism groups. OXTR levels were normalized to Hsc70, and data is presented as relative quantity. *p* = 0.089 two-tailed *t* test. **c**, **d** Spearman’s correlation coefficient and respective *p* value between miR-21-5p (**c**) and miR-451a (**d**) expression and the OXTR protein/mRNA ratio in each individual. a.u. signifies arbitrary units. There is a significant negative correlation between miR-21-5p expression and OXTR protein/mRNA ratio in human brain samples

To determine the role of miR-451a and miR-21-5p in the translation of oxytocin receptor protein, we performed Western blot on the brain samples from our current study. We detected two strong bands for OXTR at approximately 55 kD (Fig. 5a), consistent with previous findings in human and monkey brain samples [32, 33]. It is not currently clear in the literature whether these two bands are due to different isoforms or post-translational modifications. There was no significant difference in OXTR protein levels between the control and autism groups (Fig. 5b). This data is in contrast to our finding that there are significantly increased levels of OXTR mRNA in the autism brain and suggests that there may be an inhibition of protein translation.

To understand if miR-21-5p and miR-451a may have a role in inhibiting the translation of OXTR protein, we determined the protein/mRNA ratio for each sample in our analysis, and levels of miR-21-5p and miR-451a were then correlated to the protein/mRNA ratio. This analysis has previously been used to examine relationships between microRNA expression and protein translation [34]. Of great interest, there is a significant negative correlation between the expression of miR-21-5p (Fig. 5c), but not miR-451a (Fig. 5d), and the OXTR protein/mRNA ratio. This suggests that miR-21-5p may prevent the translation of OXTR protein from the existing mRNA in the human brain.

## Discussion

The findings of this study add to a growing literature exploring the molecular processes in the brain of individuals diagnosed with asd. First, the finding that miR-142 upregulation is significantly correlated with hypomethylation of the gene promoter provides further evidence of the primary role that epigenetics plays in mediation of gene expression in the autism brain. Previous studies have determined dysregulation in DNA methylation patterns in the brain [35] and lymphocytes [36] of humans with autism. Importantly, DNA methylation dysregulation was correlated with aberrant gene expression [6]. This study provides evidence for an additional mechanism for regulation of gene expression by epigenetics, where DNA hypomethylation may increase miR-142 expression, leading to possible secondary effects on downstream targets.

MiR-142 has previously been implicated in regulating proteins involved in neurotransmitter function. MiR-142-5p downregulates the transcription of monoamine oxidase A (MAOA) by decreasing the amounts of sirtuin1, a transcription factor that is involved in the transcription of MAOA [37]. MAOA metabolizes monamine neurotransmitters, including serotonin, dopamine, and noradrenaline. In addition, miR-142-3p targets and decreases the translation of D1 dopamine receptors [38]. Therefore, both miR-142-5p and miR-142-3p have important roles in the dopaminergic and monoamine pathways in the brain.

While previous studies have determined that our subset of microRNAs may target neurotransmitter-related genes, our study also identifies the oxytocin system as a target of these microRNAs. The finding that the microRNAs that can target OXTR are upregulated in the brain of individuals with autism suggests that molecular pathways responsible for social behaviors are directly targeted by microRNAs in the brain. Activation of OXTR by oxytocin is considered one of the central biological signals for social behavior. Oxytocin has been implicated in several social behaviors [28], and several independent studies have suggested that treatment of individuals with oxytocin may improve social behaviors [39–41]. Single nucleotide polymorphisms in OXTR gene have been significantly correlated to autism in a meta-analysis [29] and have also been correlated to Asperger’s syndrome in a separate study [30]. In addition, decreased plasma oxytocin levels have been reported in individuals with asd [42]. While the role of OXTR specifically in the frontal cortex has not been extensively studied, recent research determined a role for OXTR in sociosexual behavior in mice [43]. Nonetheless, further research is needed to understand the role of OXTR specifically in the frontal cortex.

The data from this research suggests an interesting interaction between miR-21-5p expression, OXTR gene expression, and OXTR protein translation. MicroRNAs can affect protein translation by degradation of the target mRNA or by inhibiting translation [44]. miR-21-5p expression positively correlated with OXTR mRNA levels, suggesting that miR-21-5p does not induce degradation of OXTR mRNA in our samples. Two recent publications have also determined positive correlations between microRNA expression in the brain and the expression levels of their target mRNAs [14, 45], including in individuals with Huntington’s disease. Therefore, we can postulate the existence of a positive feedback mechanism by which increased gene expression may induce increased microRNA expression. However, the directionality of this feedback mechanism is not clear. Of importance, the finding that miR-21-5p expression is negatively correlated with the OXTR protein/mRNA ratio suggests that miR-21-5p can inhibit the translation of OXTR and may be an important factor in limiting the levels of OXTR in the human autism brain. However, it is still problematic to conclude the function of miR-21-5p on OXTR translation through correlation data alone. Therefore, in order to strengthen and verify the conclusion that miR-21-5p is a regulator of OXTR in the brain, it will be necessary to induce an overexpression of miR-21-5p in the mouse brain, either through transgenic or lentiviral infection, and probe its effects on OXTR levels. Nonetheless, we may hypothesize that in the autism brain, attempts to increase OXTR protein levels, and downstream social behaviors, are being inhibited by miR-21-5p, thereby exacerbating the autism phenotype. Further in vivo studies may shed light onto this hypothesis.

We note that miR-451a induced a stronger downregulation of the OXTR 3′UTR activity in the luciferase assays, while only miR-21-5p was correlated with OXTR expression in the human brain. We may offer two possible hypotheses for this discrepancy. One hypothesis is that the structure of the coding region of the OXTR gene, which is not present in the luciferase plasmid, may affect the efficiency of microRNA regulation. A second hypothesis is that miR-21-5p may be expressed at higher levels in cells that express OXTR in the human brain. Since our brain samples display cell type heterogeneity, we do not know which cell types are expressing the microRNAs, and further experimentation would be necessary to understand which microRNAs are specifically expressed in OXTR expressing cells. It is also worthy to note that the previous microarray study of microRNAs in the autism brain identified increased miR-21-5p in a subset of their autism brain samples [16].

A previous study looked at microRNAs in the serum of individuals that were diagnosed with autism [15]. The microRNAs found in our study are not overlapping with those found in their study. However, both of our studies uncovered microRNAs that target genes involved in axonal guidance and TGF-beta signaling, according to the gene ontology analysis. Therefore, microRNAs involved in these pathways may be particularly sensitive to dysregulation in autism, although there may be tissue specificity in the identity of which microRNAs are dysregulated.

## Conclusions

We present evidence that microRNA dysregulation occurs in the brain of individuals diagnosed with autism spectrum disorders and that the dysregulated microRNAs target biological pathways and specific genes that are highly relevant to the biology of autism, including the OXTR gene. We also present evidence that microRNA overexpression may be mediated by epigenetic changes, which is a rather novel finding in the research of microRNAs in the brain. Therefore, this study provides further evidence of a multidimensional dysregulation of molecular mechanisms in the brain of individuals diagnosed with asd, including interplay between epigenetic mechanisms, microRNAs, and downstream mRNA targets.

## Additional files

Additional file 1: Table S1. Information on all individuals used in this analysis, including age, sex, and postmortem interval. (XLSX 10.0 KB) Additional file 2: Table S2. Raw read counts of each microRNA from each individual in the high throughput small RNA analysis. (XLSX 129 KB) Additional file 3: Table S3. Primers used in our analysis. (XLSX 9.48 KB) Additional file 4: Table S4. Correlation analysis between miR-142 expression levels and DNA methylation of miR-142 promoter. (XLSX 8.93 KB)

## Abbreviations

asd: autism spectrum disorders
ATP: Autism Tissue Program
DNA: deoxynucleic acids
OXTR: oxytocin receptor
PCR: polymerase chain reaction
RNA: ribonucleic acids

## References

[1] King BH, Navot N, Bernier R, Webb SJ (2014). Update on diagnostic classification in autism. Curr Opin Psychiatry.

[2] Persico AM, Bourgeron T (2006). Searching for ways out of the autism maze: genetic, epigenetic and environmental clues. Trends Neurosci.

[3] Hallmayer J, Cleveland S, Torres A, Phillips J, Cohen B, Torigoe T, et al. Genetic heritability and shared environmental factors among twin pairs with autism. Arch Gen Psychiatry. 2011;68:1095–102. 10.1001/archgenpsychiatry.2011.76 PMC4440679 21727249

[4] Voineagu I, Wang X, Johnston P, Lowe JK, Tian Y, Horvath S, et al. Transcriptomic analysis of autistic brain reveals convergent molecular pathology. Nature. 2011;474:380–4. 10.1038/nature10110 PMC3607626 21614001

[5] Gupta S, Ellis SE, Ashar FN, Moes A, Bader JS, Zhan J, et al. Transcriptome analysis reveals dysregulation of innate immune response genes and neuronal activity-dependent genes in autism. Nat Commun. 2014;5:5748. 10.1038/ncomms6748 PMC4270294 25494366

[6] Nardone S, Sharan Sams D, Reuveni E, Getselter D, Oron O, Karpuj M, et al. DNA methylation analysis of the autistic brain reveals multiple dysregulated biological pathways. Transl Psychiatry. 2014;4:e433. 10.1038/tp.2014.70 PMC4203003 25180572

[7] Gregory RI, Chendrimada TP, Cooch N, Shiekhattar R (2005). Human RISC couples microRNA biogenesis and posttranscriptional gene silencing. Cell.

[8] Lewis BP, Shih I, Jones-Rhoades MW, Bartel DP, Burge CB (2003). Prediction of mammalian microRNA targets. Cell.

[9] Behm-Ansmant I, Rehwinkel J, Izaurralde E (2006). MicroRNAs silence gene expression by repressing protein expression and/or by promoting mRNA decay. Cold Spring Harb Symp Quant Biol.

[10] Horman SR, Janas MM, Litterst C, Wang B, MacRae IJ, Sever MJ, et al. Akt-mediated phosphorylation of argonaute 2 downregulates cleavage and upregulates translational repression of MicroRNA targets. Mol Cell. 2013;50:356–67. 10.1016/j.molcel.2013.03.015 PMC3654076 23603119

[11] Griffiths-Jones S, Grocock RJ, van Dongen S, Bateman A, Enright AJ. miRBase: microRNA sequences, targets and gene nomenclature. Nucleic Acids Res. 2006;34(Database issue):D140–4. 10.1093/nar/gkj112 PMC1347474 16381832

[12] Geaghan M, Cairns MJ. microRNA and post-transcriptional dysregulation in psychiatry. Biol Psychiatry. 2014;78:231–239 10.1016/j.biopsych.2014.12.009 25636176

[13] Yin J, Lin J, Luo X, Chen Y, Li Z, Ma G (2014). Li K: miR-137: a new player in schizophrenia. Int J Mol Sci.

[14] Hoss AG, Kartha VK, Dong X, Latourelle JC, Dumitriu A, Hadzi TC, et al. MicroRNAs located in the Hox gene clusters are implicated in huntington’s disease pathogenesis. PLoS Genet. 2014;10:e1004188. 10.1371/journal.pgen.1004188 PMC3937267 24586208

[15] Mundalil Vasu M, Anitha A, Thanseem I, Suzuki K, Yamada K, Takahashi T, et al. Serum microRNA profiles in children with autism. Mol Autism. 2014;5:40. 10.1186/2040-2392-5-40 PMC4132421 25126405

[16] Abu-Elneel K, Liu T, Gazzaniga FS, Nishimura Y, Wall DP, Geschwind DH, et al. Heterogeneous dysregulation of microRNAs across the autism spectrum. Neurogenetics. 2008;9:153–61. 10.1007/s10048-008-0133-5 18563458

[17] Hackenberg M, Rodríguez-Ezpeleta N, Aransay AM (2011). miRanalyzer: an update on the detection and analysis of microRNAs in high-throughput sequencing experiments. Nucleic Acids Res.

[18] Dillies M-A, Rau A, Aubert J, Hennequet-Antier C, Jeanmougin M, Servant N, et al. A comprehensive evaluation of normalization methods for Illumina high-throughput RNA sequencing data analysis. Brief Bioinform. 2013;14:671–83. 10.1093/bib/bbs046 22988256

[19] Kreutz M, Hochstein N, Kaiser J, Narz F, Peist R. Pyrosequencing: powerful and quantitative sequencing technology. Curr Protoc Mol Biol. 2013;104:1–23 Unit 7.15. 10.1002/0471142727.mb0715s104 24510299

[20] Vandesompele J, De Preter K, Pattyn F, Poppe B, Van Roy N, De Paepe A, Speleman F. Accurate normalization of real-time quantitative RT-PCR data by geometric averaging of multiple internal control genes. Genome Biol. 2002;3:1–11 RESEARCH0034. 10.1186/gb-2002-3-7-research0034 PMC126239 12184808

[21] Chen A, Perrin M, Brar B, Li C, Jamieson P, Digruccio M (2015). Mouse corticotropin-releasing factor receptor type 2alpha gene: isolation, distribution, pharmacological characterization and regulation by stress and glucocorticoids. Mol Endocrinol..

[22] Elliott E, Tsvetkov P, Ginzburg I (2007). BAG-1 associates with Hsc70.Tau complex and regulates the proteasomal degradation of Tau protein. J Biol Chem.

[23] Maragkakis M, Reczko M, Simossis VA, Alexiou P, Papadopoulos GL, Dalamagas T, et al. DIANA-microT web server: elucidating microRNA functions through target prediction. Nucleic Acids Res. 2009;37(Web Server issue):W273–6. 10.1093/nar/gkp292 PMC2703977 19406924

[24] Le Belle JE, Sperry J, Ngo A, Ghochani Y, Laks DR, López-Aranda M, et al. Maternal inflammation contributes to brain overgrowth and autism-associated behaviors through altered redox signaling in stem and progenitor cells. Stem Cell Reports. 2014;3:725–34. 10.1016/j.stemcr.2014.09.004 PMC4235743 25418720

[25] Lee BK, Magnusson C, Gardner RM, Blomström S, Newschaffer CJ, Burstyn I, Karlsson H, Dalman C. Maternal hospitalization with infection during pregnancy and risk of autism spectrum disorders. Brain Behav Immun. 2014;44:100–105. 10.1016/j.bbi.2014.09.001 PMC4418173 25218900

[26] Li X, Chauhan A, Sheikh AM, Patil S, Chauhan V, Li X-M, et al. Elevated immune response in the brain of autistic patients. J Neuroimmunol. 2009;207:111–6. 10.1016/j.jneuroim.2008.12.002 PMC2770268 19157572

[27] Betel D, Wilson M, Gabow A, Marks DS, Sander C (2008). The microRNA.org resource: targets and expression. Nucleic Acids Res.

[28] Meyer-Lindenberg A, Domes G, Kirsch P, Heinrichs M (2011). Oxytocin and vasopressin in the human brain: social neuropeptides for translational medicine. Nat Rev Neurosci.

[29] LoParo D, Waldman ID. The oxytocin receptor gene (OXTR) is associated with autism spectrum disorder: a meta-analysis. Mol Psychiatry. 2014;20:640–646. 10.1038/mp.2014.77 25092245

[30] Di Napoli A, Warrier V, Baron-Cohen S, Chakrabarti B (2014). Genetic variation in the oxytocin receptor (OXTR) gene is associated with Asperger syndrome. Mol Autism.

[31] Thanseem I, Anitha A, Nakamura K, Suda S, Iwata K, Matsuzaki H, et al. Elevated transcription factor specificity protein 1 in autistic brains alters the expression of autism candidate genes. Biol Psychiatry. 2012;71:410–8. 10.1016/j.biopsych.2011.09.020 22030357

[32] Pearson SA, Mouihate A, Pittman QJ, Whelan PJ (2003). Peptidergic activation of locomotor pattern generators in the neonatal spinal cord. J Neurosci.

[33] Frayne J, Nicholson HD (1998). Localization of oxytocin receptors in the human and macaque monkey male reproductive tracts: evidence for a physiological role of oxytocin in the male. Mol Hum Reprod.

[34] Zhang S-Y, Surapureddi S, Coulter S, Ferguson SS, Goldstein JA (2012). Human CYP2C8 is post-transcriptionally regulated by microRNAs 103 and 107 in human liver. Mol Pharmacol.

[35] Ladd-Acosta C, Hansen KD, Briem E, Fallin MD, Kaufmann WE, Feinberg AP (2014). Common DNA methylation alterations in multiple brain regions in autism. Mol Psychiatry.

[36] Nguyen A, Rauch TA, Pfeifer GP, Hu VW (2010). Global methylation profiling of lymphoblastoid cell lines reveals epigenetic contributions to autism spectrum disorders and a novel autism candidate gene, RORA, whose protein product is reduced in autistic brain. FASEB J.

[37] Chaudhuri AD, Yelamanchili SV, Fox HS (2013). MicroRNA-142 reduces monoamine oxidase A expression and activity in neuronal cells by downregulating SIRT1. PLoS One.

[38] Tobón KE, Chang D, Kuzhikandathil EV (2012). MicroRNA 142-3p mediates post-transcriptional regulation of D1 dopamine receptor expression. PLoS One.

[39] Andari E, Duhamel J-R, Zalla T, Herbrecht E, Leboyer M, Sirigu A (2010). Promoting social behavior with oxytocin in high-functioning autism spectrum disorders. Proc Natl Acad Sci U S A.

[40] Guastella AJ, Einfeld SL, Gray KM, Rinehart NJ, Tonge BJ, Lambert TJ, et al. Intranasal oxytocin improves emotion recognition for youth with autism spectrum disorders. Biol Psychiatry. 2010;67:692–4. 10.1016/j.biopsych.2009.09.020 19897177

[41] Hollander E, Bartz J, Chaplin W, Phillips A, Sumner J, Soorya L, et al. Oxytocin increases retention of social cognition in autism. Biol Psychiatry. 2007;61:498–503. 10.1016/j.biopsych.2006.05.030 16904652

[42] Modahl C, Green LA, Fein D, Morris M, Waterhouse L, Feinstein C, et al. Plasma oxytocin levels in autistic children. Biol Psychiatry. 1998;43:270–7. 10.1016/s0006-3223(97)00439-3 9513736

[43] Nakajima M, Görlich A, Heintz N (2014). Oxytocin modulates female sociosexual behavior through a specific class of prefrontal cortical interneurons. Cell.

[44] BARTEL D (2004). MicroRNAs: genomics, biogenesis, mechanism, and function. Cell.

[45] Nunez YO, Truitt JM, Gorini G, Ponomareva ON, Blednov YA, Harris RA, Mayfield RD (2013). Positively correlated miRNA-mRNA regulatory networks in mouse frontal cortex during early stages of alcohol dependence. BMC Genomics.

